# Evaluation of liver fibrosis using diffusion-weighted virtual magnetic resonance elastography and ultrasound elastography

**DOI:** 10.1007/s00261-025-05043-2

**Published:** 2025-06-12

**Authors:** Mustafa Arda Onar, Mehmet Selim Nural, Aydın Deveci, Bilge Can Meydan

**Affiliations:** 1 Bartın State Hospital, Bartın, Turkey; 2 Ondokuz Mayıs University, Samsun, Turkey

**Keywords:** Diffusion magnetic resonance imaging, Chronic liver diseases, Fibrosis, Elasticity imaging techniques

## Abstract

**Introduction:**

This study evaluates the effectiveness of virtual magnetic resonance elastography (VMRE), a new diffusion-weighted imaging (DWI)-based method, for detecting liver fibrosis, comparing it with the more accessible ultrasound elastography (USE).

**Materials and methods:**

This prospective study enrolled patients with chronic liver disease who were referred for liver biopsy. Inclusion criteria were clinical indication for liver biopsy and eligibility for MRI. Exclusion criteria included MRI contraindications, hepatic iron overload, clinical or laboratory evidence of acute hepatitis or cholestasis, and inadequate image quality (motion artifacts, low signal-to-noise ratio). All patients underwent 3T VMRE (b = 200/1500 s/mm²) and two-dimensional shear wave elastography (2D-SWE). VMRE was analyzed by two blinded readers; USE by a single radiologist. Using METAVIR staging (F0-F4) as reference, fibrosis was categorized as F0-1 vs. F2-4 and F0-2 vs. F3-4. Statistical analyses included ICC, Bland-Altman, Kruskal-Wallis with Bonferroni-corrected Dunn tests, and ROC analysis. An HBV subgroup (*n* = 33) and a non-HBV group (*n* = 16; including metabolic dysfunction-associated steatotic liver disease (MASLD), autoimmune, and toxic hepatitis) were analyzed separately to assess VMRE performance across different etiologies.

**Results:**

Initially, 59 patients were enrolled. After excluding 10 patients due to MRI contraindications, hepatic iron overload, or inadequate image quality, 49 patients were included in the final analysis (mean age 48.2 ± 14.9 years; 28 males, 21 females; 67% HBV-positive). VMRE demonstrated significant limitations in clinical utility, failing to discriminate fibrosis stages in the overall cohort (AUC 0.45–0.51, *p* > 0.05). While HBV-infected patients showed some promise with an overall significant variation across stages (*p* = 0.004), post-hoc analysis revealed VMRE could only distinguish between the extreme ends of the fibrosis spectrum (F0 vs. F4: adjusted *p* = 0.0058). This restricted diagnostic capability was reflected in the HBV subgroup’s modest AUC values of 0.75–0.76, which remained below clinical acceptability thresholds. In striking contrast, ultrasound elastography exhibited robust performance across all analyses. It achieved excellent diagnostic accuracy (AUC 0.86–0.95) with highly significant p-values (< 0.001) for all fibrosis classifications, along with clinically practical threshold values (8.85–10.1 kPa). Inter-rater agreement for VMRE was excellent (ICC = 0.972), and intra-rater agreement for USE was good (ICC = 0.756).

**Conclusion:**

VMRE demonstrates insufficient diagnostic accuracy for fibrosis staging in both HBV and non-HBV populations, with only limited ability to distinguish extreme fibrosis stages (F0 vs. F4) in HBV patients. While showing excellent technical reproducibility (ICC = 0.972), its poor discriminative performance (AUC 0.45–0.76 across groups) and inability to differentiate intermediate stages preclude clinical utility. In contrast, USE achieved consistently superior diagnostic accuracy (AUC 0.86–0.95) with practical threshold values, supporting its preference over VMRE especially in centers lacking access to standard MRE. Further VMRE development requires technical optimization and larger validation studies.

## Introduction

Chronic liver disease (CLD) represents a major global health burden, with etiology varying by region: metabolic dysfunction-associated steatotic liver disease (MASLD) predominates in developed nations, while hepatitis B (HBV) and C (HCV) infections remain prevalent in developing regions [1, 2]. Fibrosis, a key marker of liver function, is linked to severe complications like portal hypertension and hepatocellular carcinoma [3]. Early diagnosis and accurate staging of fibrosis are crucial, as recent studies suggest fibrosis may be reversible with timely treatment [4]. Although liver biopsy remains the histological gold standard for fibrosis staging, its use in longitudinal monitoring is limited by key constraints: invasive procedural risks, sampling variability due to heterogeneous fibrosis distribution, and high patient refusal rates [5–7]. Additionally, liver biopsy is not ideal for repeated follow-up due to its invasive nature and associated risks. These limitations have accelerated the shift toward non-invasive methods. While serum markers like FibroTest and aspartate aminotransferase to platelet ratio index (APRI) are commonly used, their moderate sensitivity and tendency for false positives restrict their reliability [8]. This gap highlights the need for more accurate and reproducible tools suitable for serial assessment, a role that emerging imaging techniques may help fulfill.

Ultrasound elastography (USE), including techniques such as two-dimensional shear wave elastography (2D-SWE), is increasingly used for liver fibrosis assessment due to its non-invasive nature and accessibility ccording to meta-analyses, USE demonstrates high diagnostic performance for detecting significant fibrosis (≥ F2), with pooled sensitivity ranging from 85 to 88% and specificity between 79% and 91%, depending on fibrosis stage and study population​ [9–12]. However, the diagnostic accuracy of USE can be limited by operator dependence, variability in device-specific cutoff values, and inter-system reproducibility issues [13]. Magnetic resonance elastography (MRE) has emerged as the most accurate non-invasive imaging modality for liver fibrosis assessment. By combining mechanical wave propagation with MRI-based phase contrast imaging, MRE enables quantitative measurement of liver stiffness over a large hepatic volume. Magnetic resonance elastography (MRE) has consistently demonstrated high diagnostic performance for assessing liver fibrosis. Meta-analyses report pooled sensitivity ranging from 88 to 94% and specificity between 89% and 95% for detecting significant fibrosis (≥ F2), with AUROC values ranging from 0.88 to 0.98 [12, 14, 15]. However, its broader clinical application is limited by higher costs, longer acquisition times, and a greater number of contraindications compared to ultrasound-based techniques, including issues related to claustrophobia, implanted devices, and reduced patient compliance. [16, 17]. Diffusion-weighted imaging (DWI) has also been explored for fibrosis assessment, showing an inverse relationship between ADC values and fibrosis severity, though its diagnostic accuracy is moderate compared to MRE [18, 19].

Recent advances include Virtual Magnetic Resonance Elastography (VMRE), which converts shifted ADC values derived from diffusion-weighted imaging into tissue elasticity measures. VMRE has shown a high correlation with conventional MRE and potential in distinguishing fibrosis stages [20, 21]. Compared to conventional MRE, VMRE offers potential advantages in terms of cost-effectiveness and accessibility, as it does not require external mechanical hardware and can be performed using routine DWI sequences available in most clinical MRI protocols. However, it is still in the research phase and has not yet been approved for routine clinical use. Moreover, limited studies have evaluated its reproducibility and diagnostic performance across different MRI platforms and patient populations.This study aims to evaluate VMRE’s effectiveness in assessing liver fibrosis across diverse patient populations and devices and to compare its performance with USE.

## Materials and methods

This prospective, single-center study, approved by the local institutional review board, involved adult patients who were referred to our Hepatology Clinic for liver biopsy and were eligible. All ultrasound elastography (USE) examinations were systematically performed before the liver biopsy procedure as part of the routine pre-biopsy clinical assessment. Similarly, all MRI examinations for VMRE acquisition were completed prior to the liver biopsy, within a standardized interval of no more than 30 days (median: 7 days; range: 1–28 days). At the time of imaging, histopathological results were not yet available. Operators performing USE and readers evaluating VMRE images were blinded to biopsy and clinical data, ensuring that both acquisition and interpretation were conducted without bias. Inclusion criteria included age ≥ 18 years, referral for liver biopsy due to suspected or known chronic liver disease, and eligibility for liver MRI. Exclusion criteria included contraindications to MRI (e.g., pacemakers, metallic implants, claustrophobia), clinical or laboratory evidence of acute hepatitis or cholestasis, hepatic hemosiderosis, and inadequate quality due to artifacts such as low signal-to-noise ratio, motion artifacts, or image distortion.

### Histopathological examination

Liver biopsies were performed under local anesthesia using an ultrasound-guided approach targeting the right hepatic lobe. A semi-automated 18-gauge Tru-Cut biopsy needle was used in all cases. Adequate tissue samples were obtained, with all biopsy specimens containing at least 11 portal tracts to ensure diagnostic reliability. In this study, histopathological evaluation of the presence and degree of hepatic fibrosis and inflammation was accepted as the standard reference. All histological specimens were scored by hepatobiliary pathologists with 16 years of experience. All biopsy samples were stained with hematoxylin-eosin and Masson trichrome. Fibrosis was staged using the METAVIR scoring system (F0-F4), which served as the reference standard for both VMRE and USE. (22). The stages of fibrosis were defined as follows: F0, no fibrosis; F1, portal fibrosis without septa formation; F2, portal fibrosis with a few septa; F3, numerous septa without cirrhosis; F4, cirrhosis. Based on this, patients were categorized into significant and non-significant fibrosis (F0-1 vs. F2-4) and early-stage vs. advanced fibrosis (F0-2 vs. F3-4).

### Imaging protocol

All MRI examinations were performed using a 3T scanner (Philips Ingenia^®^, Best, Netherlands) with a 16-channel body array coil. Diffusion-weighted imaging (DWI) was acquired using a single-shot echo-planar imaging (ss-EPI) sequence with a free-breathing technique. Detailed acquisition parameters are summarized in Table 1.

**Table 1:** MRI acquisition parameters for diffusion-weighted imaging

Parameter	Value
Sequence type	ss-EPI
Breathing technique	Free-breathing
TR / TE (ms)	3000 / 78
b-values (s/mm²)	200 and 1500
Number of averages	b = 200 → 4; b = 1500 → 8
Field of view (FOV, mm)	360 × 360 to 400 × 400
Matrix size	80 × 100
Slice thickness / gap (mm)	5 / 1
Total scan time	4 min 30 s

USE was performed by a radiology resident with 5 years of experience using the 2D-SWE technique on the Mindray DC-40 (Mindray, Shenzhen, China) device. Measurements were taken from the right lobe in a mild right lateral decubitus position with the right arm maximally abducted, and patients were instructed to maintain a neutral breathing state. USE images were generated on a gray-scale ultrasound background.

To assess intraobserver reproducibility, two independent acquisitions were performed consecutively for each patient by the same radiology resident. In each acquisition, two circular ROIs (10 mm diameter) were manually placed by a single radiologist with 5 years of experience, within the same elasticity color box on an axial image, avoiding large vessels and bile ducts, and ensuring a minimum depth of 2 cm from the liver capsule. Most measurements were obtained at depths between 2 and 5 cm, while in cases such as obesity, measurements up to 6 cm were accepted provided that image quality was adequate. The mean value of the two ROIs from each acquisition was recorded, and the mean of the two acquisitions was used for final analysis. All measurements adhered to an interquartile range (IQR) to median ratio (IQR/median) ≤ 30%, according to international elastography guidelines [23].

### Image analysis

The shifted ADC was calculated from DW MRI signals acquired with two optimal b-values of 200 and 1500 s/mm², using the formula:

$$ {\text{sADC (mm2/s) = ln(S200/S1500)/1300}} $$

where sADC is the shifted ADC, S200 is the DW MRI signal with a b-value of 200 s/mm², and S1500​ is the DWI MR signal with a b-value of 1500 s/mm². VMRE value (kPa) was then calculated as:

$$ {\text{VMRE (kPa)}}\,{\text{ = }}\alpha {\text{ sADC (mm}}^{{\mathrm{2}}} {\text{/s) + }}\beta $$

**Fig. 1:**
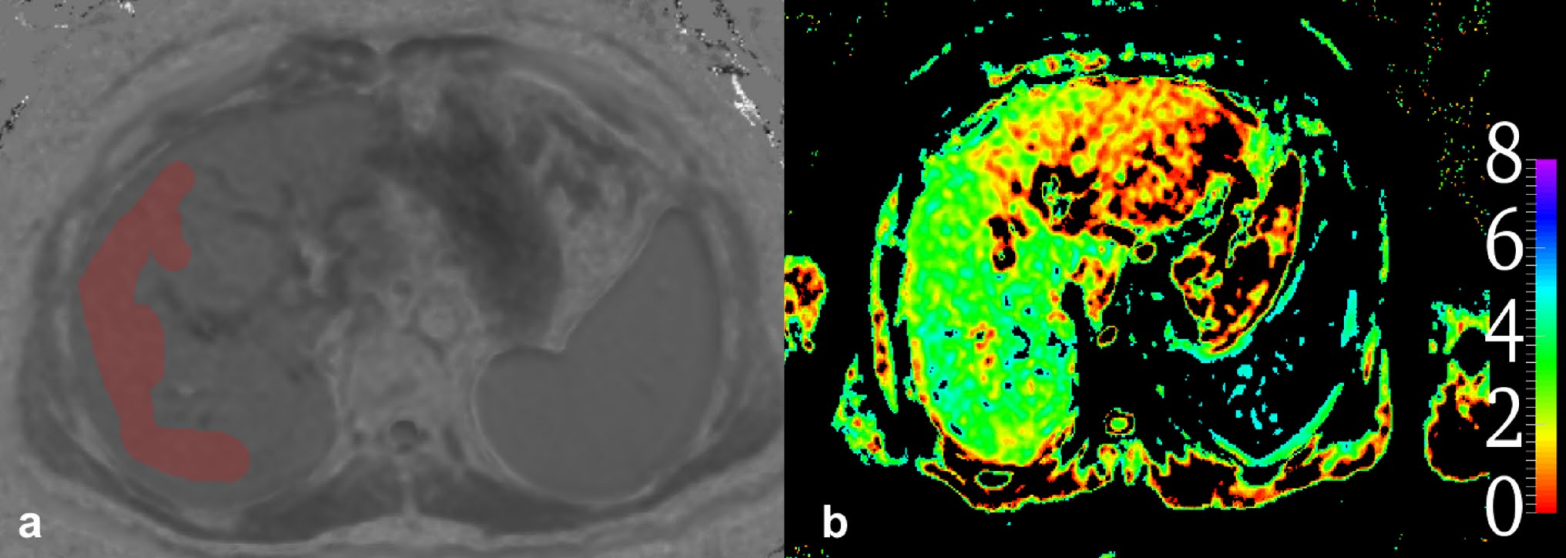
VMRE color map **a**, VMRE segmentation **b**

The values for α and β (-12.740 and 14.0) reported by Le Bihan et al., which correlate the results with standard MRE, were used from the calibration study reported earlier in a different patient cohort [20]. Diffusion weighted images were processed using FSLeyes software to generate VMRE color map (Fig. 1b) [24]. An abdominal radiologist with 15 years of experience and a radiology resident with 5 years of experience independently analyzed the images of 49 patients. Both observers were blinded to histopathological results and clinical data during the image evaluation to avoid interpretation bias. They drew ROIs, excluding artifacts and vascular structures, on at least three consecutive sections of the right liver lobe using ITK-SNAP 4.0 software shown in (Fig. 1a) [25].

### Statistical analysis

Statistical analyses were performed using SPSS software version 21.0 (Chicago, Illinois, USA). Prior to patient enrollment, an a priori power analysis was conducted using G*Power software. The analysis indicated that a minimum of 40 patients would be required to detect medium effect sizes (Cohen’s d = 0.5) with a power of 80% and a two-sided alpha level of 0.05. Our final sample included 49 patients, exceeding this threshold. The normality of continuous variables was assessed using the Kolmogorov–Smirnov test, with a p-value greater than 0.05 indicating normal distribution. The reliability of the VMRE and USE measurements was evaluated using intraclass correlation coefficients (ICCs), calculated as interobserver agreement for VMRE and intraobserver agreement for USE. Descriptive statistical analyses, including mean, standard deviation (SD), and frequency, were used for parametric data, while the Kruskal-Wallis test was used for non-parametric data, and the one-way ANOVA test was used for parametric comparisons. When the Kruskal-Wallis test revealed statistical significance, Bonferroni-adjusted Dunn post hoc tests were applied to determine which fibrosis stages differed significantly.

In this study, all cases with liver disease of various etiologies and the HBV subgroup were analyzed separately. Receiver operating characteristic (ROC) curve analysis was used to determine the optimal threshold values for VMRE and USE. The optimal cut-off points were determined using the Youden index. Diagnostic sensitivity, specificity, positive predictive value (PPV), negative predictive value (NPV), and accuracy were calculated. A p-value < 0.05 was considered statistically significant within a 95% confidence interval. Additionally, the diagnostic effectiveness of VMRE was evaluated by comparing the threshold values obtained in this study with previously published standard MRE thresholds and histopathological results [26].

## Results

### Patient characteristics

A total of 59 patients were included in the study. After excluding 2 patients with hemosiderosis and 8 due to low image quality, 49 patients (28 males 57.1% and 21 females 42.9%) were included in the final analysis. The demographic information, causes of CLD, and laboratory values of the patients included in the study are presented in Table 2. The mean age of the patients was 48.16 ± 14.89, with an age range of 19–74 years. The mean VMRE measurements were 4.53 ± 1.37 kPa, ranging from 2.1 to 9.6 kPa.

**Table 2:** Demographic, etiologic, and laboratory characteristics of patients stratified by METAVIR fibrosis stage

METAVIR Stage	*n*	Male/Female (*n*)	Age (Mean ± SD)	HBV (%)	MASLD (%)	Other (%)	AST (U/L)	ALT (U/L)	PLT (×10^3/µL)
F0	8	2/6	44.0 ± 16.91	87.5	12.5	0	27.62 ± 16.26	35.12 ± 45.01	237.5 ± 60.33
F1	13	4/9	46.69 ± 14.95	56.2	12.5	31.2	70.15 ± 62.61	88.74 ± 95.71	250.61 ± 52.50
F2	6	5/1	48.67 ± 12.24	83.3	0	16.7	80.83 ± 75.41	59.67 ± 46.13	151.67 ± 37.23
F3	12	8/4	51.58 ± 17.90	66.7	16.7	16.7	48.0 ± 28.27	50.67 ± 52.09	160.25 ± 57.0
F4	7	7/0	50.0 ± 10.61	57.1	14.3	28.6	42.43 ± 14.81	46.71 ± 24.78	146.57 ± 82.23

*Other CLD” includes autoimmune hepatitis (n = 3), toxic hepatitis (n = 2), and non-hepatitis-related cirrhosis (n = 2). Three patients without established chronic liver disease (n = 3) in the F1 stage are not represented in subgroup percentages but were included in all imaging and statistical analyses. Subgroup percentages in F1 were calculated based on patients with established chronic liver disease (n = 13).

### Interreader agreement

**Fig. 2:**
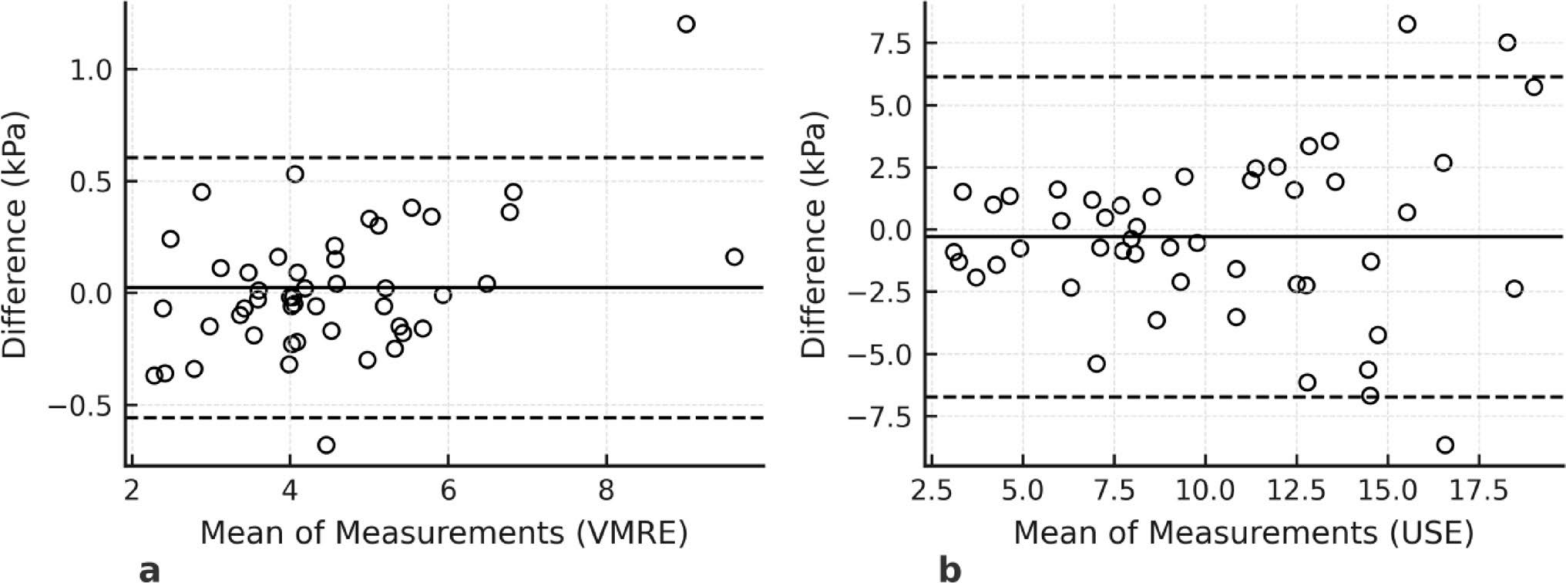
Bland–Altman plots of interrater agreement for VMRE (**a**) and USE (**b**) dashed lines are mean and ± 1 standard deviation (UL = upper limit; LL = lower limit)

The Bland-Altman plots showing the agreement between the two independent observers for VMRE measurements and the two separate measurements performed by a single observer for USE are presented in Fig. 2.The mean difference and standard deviation between the observers were calculated as 0.022 and 0.299 for VMRE, and 0.035 and 3.3 for USE, respectively. The ICC was calculated to be 0.972 (95% CI: 0.953–0.987) for VMRE and 0.756 (95% CI: 0.598–0.853) for USE, indicating excellent interobserver agreement for VMRE and good intraobserver agreement for USE.

### Comparison of VMRE and USE measurements across fibrosis stages

**Fig. 3:**
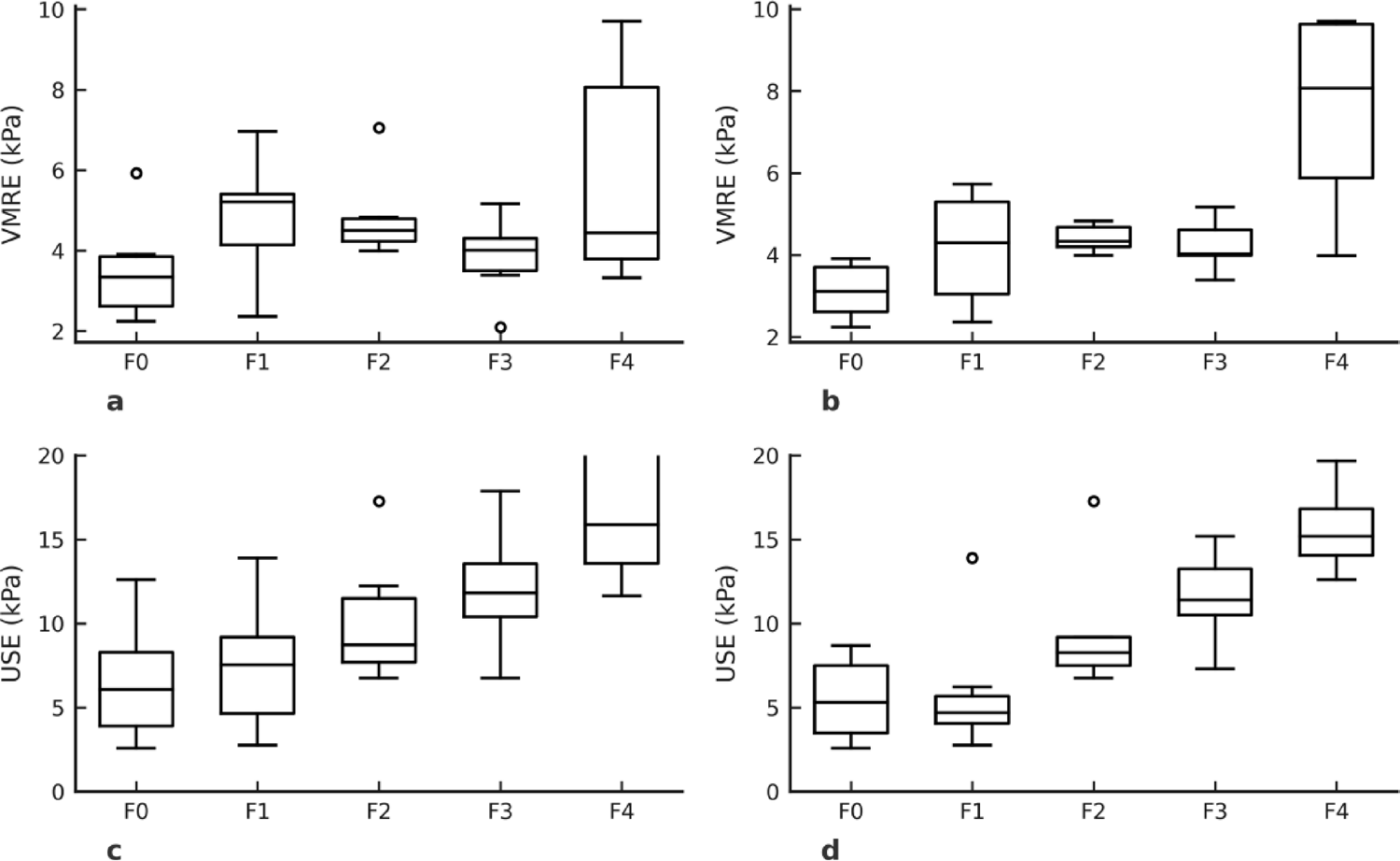
Box plots of VMRE of all patients **a**, VMRE of HBV patients **b**, USE of all patients **c** and USE of HBV patients **d**

The averages of the VMRE values (kPa) measured by the two observers and the mean of two separate USE (kPa) measurements obtained by a single observer were used for statistical analyses. The VMRE (kPa) and USE (kPa) values of the cases according to fibrosis stages are shown in Tables 3 and 4. The differences in VMRE values between fibrosis stages were analyzed using the Kruskal-Wallis test, and a statistically significant difference was found between these values (*p* = 0.016). In the HBV group, the difference between fibrosis stages was found to be statistically stronger (*p* = 0.004). Post hoc analysis using the Dunn test with Bonferroni correction revealed that among all patients pairwise comparisons were non-significant. In the HBV subgroup, the Dunn test demonstrated significant differences between F0 and F4 (*p* = 0.0058), suggesting slightly better stage discrimination in this subgroup. However, other pairwise comparisons within the HBV group did not reach statistical significance. (Table 4) The difference in USE measurements between fibrosis stages was evaluated using the One-Way ANOVA test, and a statistically significant difference was found between the stages (*p* < 0.001). The distribution of VMRE (kPa) and USE (kPa) according to fibrosis staging in all cases and the HBV subgroup is shown in the box plot curves. ( Fig. 3)

**Table 3:** Median ± IQR sADC (mm2/s) and mean ± sd (Min–Max) USE (kPa) values

ADC (mm²/s)	F0		F1		F2		F3		F4	
All Patients	0.836 (0.753–0.893)		0.69 (0.675–0.774)		0.742 (0.721–0.766)		0.804 (0.771–0.832)		0.786 (0.669–0.826)	
HBV Patients	0.855 (0.805–0.893)		0.694 (0.682–0.852)		0.758 (0.726–0.768)		0.784 (0.771–0.832)		0.687 (0.524–0.793)	
US Elastography (kPa)	F0		F1		F2		F3		F4	
All Patients	6.0 ± 4.11 (2.59–12.61)		8.74 ± 4.18 (2.75–21.07)		10.74 ± 3.57 (6.75–17.28)		13.68 ± 4.18 (8.77–22.03)		18.05 ± 4.84 (12.61–21.87)	
HBV Patients	4.35 ± 2.09 (2.59–6.84)		8.07 ± 4.57 (2.75–21.07)		8.88 ± 2.62 (6.75–14.52)		11.55 ± 2.61 (8.77–15.19)		16.13 ± 4.99 (12.61–19.66)	

**Table 4:** Median (IQR) VMRE values across METAVIR stages, with Kruskal-Wallis and dunn test results (Bonferroni-adjusted)

Group	*n*	Stage	Median (kPa)	IQR (kPa)	*P* value	Significant dunn comparisons (*p* < 0.05)
All Patients (*n* = 49)					0.016	
	8	F0	3.28	2.65–4.14		
	16	F1	5.21	4.28–5.47		
	6	F2	4.32	4.08–4.96		
	12	F3	4.03	3.60–4.26		
	7	F4	6.51	3.90–7.29		
HBV Patients (n:33)					0.004	F0 vs. F4 (*p* = 0.0058)
	7	F0	3.17	2.80–3.50		
	9	F1	4.12	3.90–5.30		
	5	F2	4.28	4.20–4.60		
	8	F3	4.20	3.90–4.50		
	4	F4	6.93	6.50–8.70		

### Diagnostic performance of VMRE and USE

**Fig. 4:**
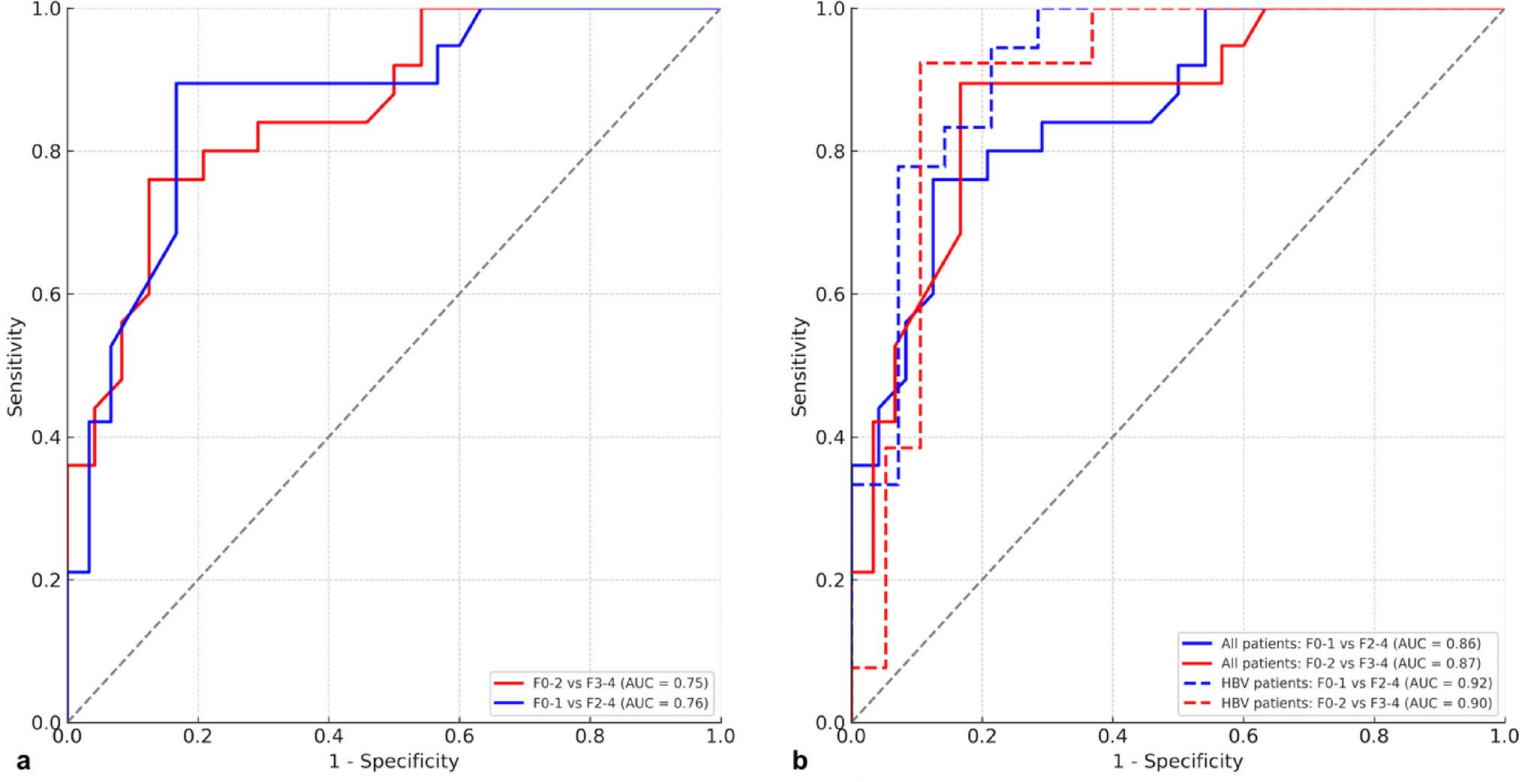
Receiver operating characteristic (ROC) curves of VMRE (**a**) and USE (**b**) for differentiating fibrosis stages F0-1 vs. F2-4 and F0-2 vs. F3-4. The curves in (**a**) represent HBV patients only, while (**b**) includes both all patients and the HBV subgroup

The diagnostic efficacy of VMRE and USE was evaluated based on histopathological staging. The corresponding diagnostic thresholds, sensitivities, and specificities for each fibrosis classification are summarized in Table 5. Although only the F0-F4 comparison reached statistical significance in post hoc analysis, ROC analyses were conducted for clinically relevant binary groupings (F0-1 vs. F2-4 and F0-2 vs. F3-4), as these thresholds align with standard fibrosis staging cutoffs used in treatment decision-making. This approach enabled evaluation of each modality’s potential diagnostic utility in practice, beyond individual stage-wise comparisons.The validity of VMRE values in distinguishing between significant and non-significant fibrosis (F0-1 vs. F2-4) and early versus advanced stages (F0-2 vs. F3-4) was assessed using ROC curve analysis. Across all cases, the AUC values for distinguishing between significant and non-significant fibrosis (F0-1 vs. F2-4) and early versus advanced fibrosis (F0-2 vs. F3-4) were 0.51 and 0.45, respectively, indicating that VMRE did not demonstrate a discriminative performance in distinguishing fibrosis stages. In the HBV group, a sensitivity of 84% and specificity of 64% were achieved for distinguishing between F0-F1 and F2-F4 stages at a threshold value of 3.98 kPa (AUC = 0.76). For distinguishing between F0-F2 and F3-F4 stages in the HBV subgroup, a sensitivity of 80% and specificity of 67% were obtained at a threshold value of 4.04 kPa (AUC = 0.75) (Fig. 4a). These results suggest that VMRE measurements are applicable in the HBV group.

**Table 5:** Receiver operating characteristics for measured data with the area under the receiver operating characteristic curves (AUROCs)

Comparison	Test	Sensitivity (%)	Specificity (%)	AUC	Optimal Cut Off	PPV (%)	NPV (%)	*p* value
F0-1 versus F2-4 in HBV patients	VMRE (kPa)	84	64	0.76 (0.55–0.93)	3.98	76	75	0.013
F0-2 versus F3-4 HBV patients	VMRE (kPa)	80	67	0.75 (0.56–0.90)	4.04	67	80	0.022
F0-1 versus F2-4 in all patients	USE (kPa)	76	88	0.86 (0.76–0.96)	8.98	86	77	< 0.001
F0-1 versus F2-4 in HBV patients	USE (kPa)	94	83	0.92 (0.81–0.98)	7.30	85	91	< 0.001
F0-2 versus F3-4 in all patients	USE (kPa)	90	86	0.90 (0.79–0.98)	9.83	81	91	< 0.001
F0-2 versus F3-4 in HBV patients	USE (kPa)	93	94	0.95 (0.86–0.99)	10.05	93	94	< 0.001

**Fig. 5:**
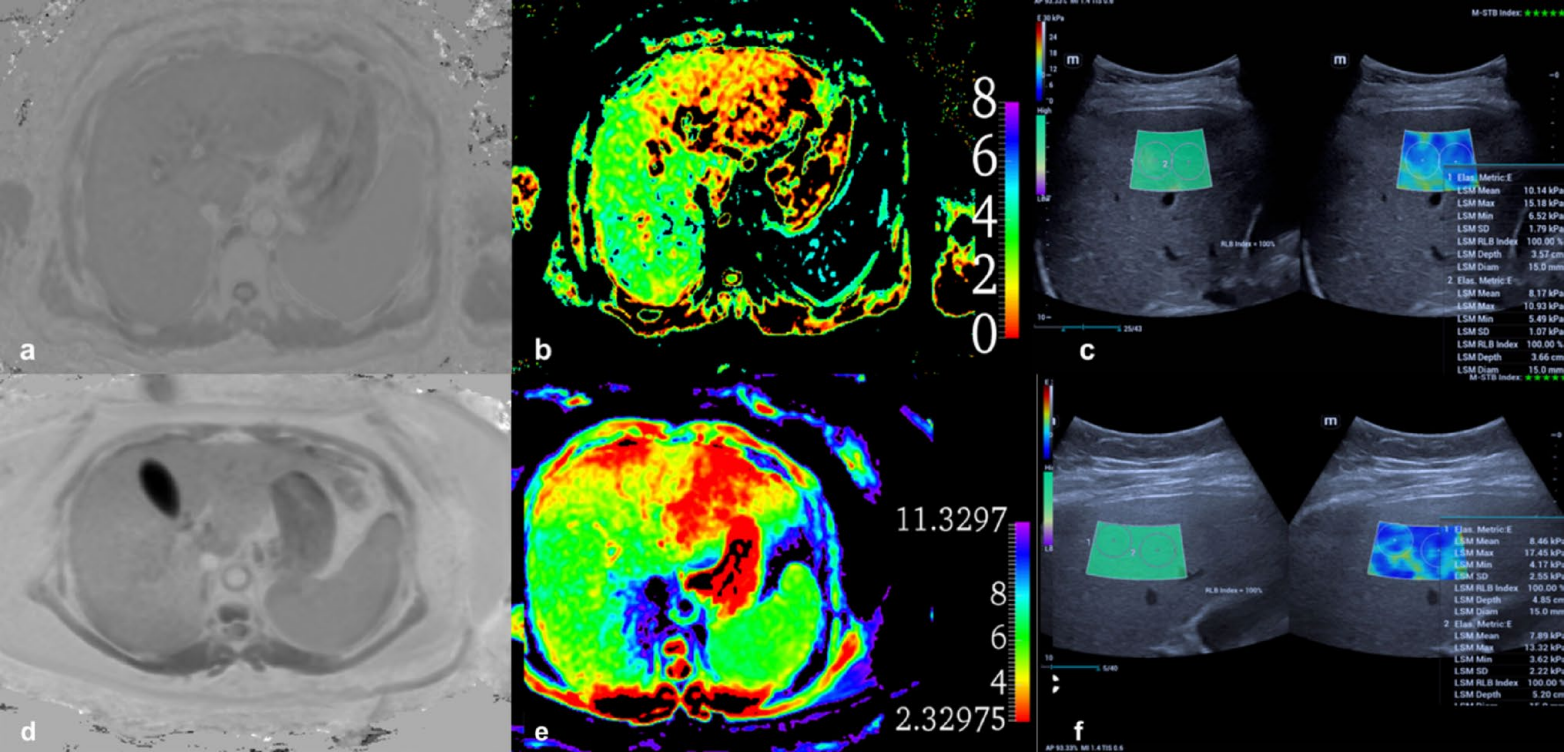
A 48-year-old male patient diagnosed with Hepatitis B, with a fibrosis score of F2. VMRE images (**a**), color map (**b**), and USE image (**c**). The VMRE value was calculated as an average of 3.68 kPa. In the USE measurements, the SW value was 9.73 kPa, indicating that the measurements made by both USE and VMRE were consistent with the patient’s pathological stage. A 51-year-old female patient diagnosed with MASLD, with a fibrosis score of F1. VMRE images (**d**), color map (**e**), and USE (**f**) image. the VMRE value was calculated as an average of 6.2 kPa, while the SW value in the USE measurement was found to be7.68 kPa. the VMRE measurements were inconsistent with the pathological stage, whereas the USE measurements were consistent with the pathological stage

The validity of USE in distinguishing between significant and non-significant fibrosis (F0-1 vs. F2-4) and early versus advanced stages of fibrosis (F0-2 vs. F3-4) was also assessed using ROC curve analysis. Across all cases, a sensitivity of 76% and specificity of 88% were obtained for distinguishing between F0-1 and F2-4 stages at a threshold value of 8.98 kPa (AUC = 0.86). For distinguishing between F0-F2 and F3-F4 stages, a sensitivity of 90% and specificity of 86% were achieved at a threshold value of 9.83 kPa (AUC = 0.90) (Fig. 4b). Representative patient cases are shown in Fig. 5.

### Comparison of VMRE-based fibrosis staging with standard MRE Cut-Off values

In our study, liver stiffness values obtained from VMRE were classified according to the standard fibrosis stage thresholds established for conventional MRE by Morisaka et al., which correspond to the METAVIR scoring system [26]. These thresholds were also adopted by Kromrey et al. in the initial validation of VMRE, and were used to categorize fibrosis stages in our cohort. The standard fibrosis stage thresholds are summarized in Table 6. In the full cohort, fibrosis stage matched exactly in 13 patients (27%) and within one stage in 26 patients (53%). In the HBV subgroup, exact matches were observed in 9 cases (27%), and one-stage differences in 19 cases (58%). When analyzed using binary classifications, concordance for significant vs. non-significant fibrosis (F0-1 vs. F2-4) was found in 28 of 49 patients (57%) overall and 23 of 33 HBV patients (75%). For early-stage vs. advanced fibrosis (F0-2 vs. F3-4), the concordance was 24 of 49 patients (49%) overall and 22 of 33 HBV patients (72%). Compared to the fixed MRE thresholds, the ROC-derived thresholds based on our histopathological findings yielded higher concordance rates, particularly within the HBV subgroup. This suggests that data-driven thresholds may improve classification accuracy over literature-based fixed cut-offs in select populations.

**Table 6:** Standard MRE fibrosis thresholds used for VMRE categorization [26]

Fibrosis stage	Mean (kPa)	Standard deviation (kPa)
F0	2.13	0.27
F1	2.49	0.28
F2	2.83	0.32
F3	3.6	0.81
F4	5.2	1.02

## Discussion

This study compared the diagnostic performance of VMRE and USE for liver fibrosis staging using histopathological METAVIR scores as the gold standard. The results revealed significant limitations in VMRE’s clinical utility. While the technique showed statistically significant variation across fibrosis stages in the HBV subgroup (*p* = 0.004), post-hoc analysis with Bonferroni correction demonstrated that VMRE could only reliably distinguish between the extreme ends of the fibrosis spectrum (F0 vs. F4, adjusted *p* = 0.0058). No significant differentiation was observed between intermediate stages in either the full cohort or HBV subgroup. ROC curve analysis confirmed these findings. In the overall population, VMRE showed poor diagnostic performance with AUC values of 0.51 for distinguishing F0-1 from F2-4 stages and 0.45 for differentiating F0-2 from F3-4 stages. While performance improved somewhat in the HBV subgroup (AUC 0.75–0.76), these values remained below the 0.80 threshold generally considered clinically acceptable. This approach enabled evaluation of VMRE’s clinical applicability, despite its inability to consistently differentiate between all fibrosis stages. The standard binary classifications (F0-1 vs. F2-4 and F0-2 vs. F3-4) were specifically chosen for ROC analysis because they represent clinically meaningful thresholds that guide treatment decisions in practice. This evaluation framework provides the most relevant assessment of real-world diagnostic utility, even when a technique cannot discriminate all individual stages. The findings suggest VMRE may have limited value in identifying cirrhosis (F4) in HBV patients, but its poor performance in staging intermediate fibrosis - particularly the crucial distinction between F2 and F3 stages - significantly restricts its clinical usefulness compared to established methods like USE, which demonstrated superior performance across all analyses.

In the study by Kromrey et al., which evaluated VMRE, the measurements were assessed by referencing the standard MRE values reported in the literature [21]. It was demonstrated that VMRE had a sensitivity of 76% and specificity of 65% (AUC: 0.72) for distinguishing between F0-1 and F2-4 stages with a cutoff value of 3.10 kPa, and a sensitivity of 91% and specificity of 67% (AUC: 0.79) for distinguishing between F0-2 and F3-4 stages with a cutoff value of 3.29 kPa. The effectiveness of VMRE in the HBV group in our study appears to be consistent with the literature. The stronger distinction in the HBV group may be due to the differential impact of steatosis and other inflammatory processes on ADC values. Studies in the literature that examine the effect of hepatic steatosis on diffusion parameters have reported varying results. Bonekamp et al. did not find a clear relationship between ADC and steatosis, while Harada et al. observed a strong correlation between ADC and severe steatosis [18, 27] Hannimal et al., in their study of 49 MASLD patients, modified the VMRE equation by calculating the fat fraction and reported no correlation between VMRE values and fibrosis [28]. In our study, when only the HBV group was considered excluding cases of MASLD and non-viral hepatitis, VMRE demonstrated moderately improved performance in distinguishing fibrosis stages. This relative improvement may be linked to reduced interference from hepatic fat, as factors such as fat-related effects on ADC, low signal-to-noise ratio at high b-values, inadequate fat suppression, and challenges in magnetic field homogeneity (especially in patients with higher BMI) may have compromised VMRE accuracy in non-HBV patients. The VMRE equation used in our study with b-values of 200–1500 was adapted from the study by Kromrey et al., where the majority of the cases (77%) were viral hepatitis patients [21]. Therefore, in non-viral hepatitis cases, a different correlation may be necessary to obtain VMRE values. Ultimately, it should be remembered that the reliability of VMRE in patients with high BMI may be compromised due to various factors, and results should be interpreted accordingly.

In the initial validation of VMRE, Kromrey et al. applied the standardized MRE thresholds established by Morisaka et al. to classify fibrosis stages [26]. To maintain methodological consistency with prior VMRE studies and to facilitate comparison with published results, we similarly adopted Morisaka’s thresholds for fibrosis stage categorization in our analysis. In the study by Kromrey et al., the exact matching rate for fibrosis staging using VMRE based on these standard MRE thresholds was 55%, with a 90% agreement within a one-stage difference. In comparison, in our study, the exact matching rate was 27%, and the one-stage difference agreement was 53%. In the HBV subgroup, these rates were 30% and 63%, respectively (21). Our rates are lower compared to those reported by Kromrey et al. Kromrey et al. performed DWI with breath-triggered imaging, whereas we used a free-breathing technique due to technical reasons. In the study by Kwee et al., which compared free-breathing and breath-triggered imaging, ADC values were found to be approximately 0.4–0.5 units higher in breath-triggered imaging compared to other methods [29]. The higher VMRE values and threshold values in our study, which are inversely proportional to sADC values, may be attributed to the imaging technique used. Additionally, Kromrey et al. performed voxel-based analysis and excluded values with a standard deviation greater than 15%, whereas we selected only artifact-free regions in our ROI selection. This could be another reason for the lower matching rates and higher optimal threshold values in our study. When fibrosis staging using VMRE in our study was performed based on histopathological results rather than the standard MRE data from the literature, higher concordance and rates were observed in distinguishing fibrosis stages. This suggests that using customized threshold values tailored to the device and protocols may be more appropriate for VMRE in distinguishing fibrosis stages.

In the recent study by Wang et al., involving 81 cases and various b-value pairs, it was found that ADC values obtained with b-values of 200–800 and 200–1000 provided better differentiation of fibrosis stages compared to other b-value pairs, and these ADC values correlated better with standard MRE [30]. Furthermore, in this study, the ADC values obtained with the b-values of 200–1500, which were also used in our study, were found to show statistically significant differences between fibrosis stages (*p* < 0.001), but, as in our study, were insufficient in distinguishing between fibrosis stages. The reasons for these differing results in the literature may be due to the differential impact of fibrosis on ADC depending on the underlying etiology, as well as differences in equipment and b-values. There is no standard protocol or b-value set for DWI, and more extensive studies involving various b-values and different equipment are needed to obtain more reliable information in this area.

Furthermore, all patients in our study underwent USE examination, which proved to be more reliable than VMRE. USE showed a sensitivity of 76% and specificity of 88% (AUC: 0.86) for distinguishing F0-1 from F2-4 stages, and 90% and 86% (AUC: 0.90) for differentiating F0-2 from F3-4 stages. These findings are consistent with meta-analyses by Zhang et al. and Luo et al., further validating USE’s efficacy in fibrosis staging [31, 32]. Similar results were obtained in studies evaluating MASLD cases, demonstrating USE’s applicability across various liver disease etiologies. Given these outcomes, USE appears to be more reliable and suitable than VMRE for fibrosis diagnosis and staging, especially in centers lacking access to standard MRE.

### Limitations

This study has several limitations. First, while our overall sample size (*n* = 49) is comparable to prior VMRE studies and provides adequate statistical power for primary analyses, the HBV subgroup (*n* = 33) remains relatively small, requiring cautious interpretation of these specific results. Second, although free-breathing acquisition introduced motion artifacts in 8 patients (13.3%), we minimized their impact through careful manual ROI selection in artifact-free regions. Third, the uneven distribution of fibrosis stages (particularly underrepresentation of F2) necessitated binary classification for robust analysis. Fourth, unavailable clinical variables (e.g., BMI, detailed treatment history) may represent confounding factors. Finally, inter-observer reliability was excellent for VMRE (ICC = 0.972), intra-observer and test-retest reproducibility warrant future evaluation.

## Conclusion

VMRE, though promising, currently lacks sufficient diagnostic efficacy for staging fibrosis across different etiologies. The better results obtained in the HBV subgroup suggest the need for further studies focusing on etiology-specific effectiveness. In contrast, USE, supported by extensive literature, remains a reliable method for diagnosing and staging fibrosis and can be effectively used in place of VMRE in centers lacking standard MRE.

## Data Availability

No datasets were generated or analysed during the current study.
