# Correction: Evaluation of liver fibrosis using diffusion-weighted virtual magnetic resonance elastography and ultrasound elastography

**DOI:** 10.1007/s00261-026-05427-y

**Published:** 2026-03-24

**Authors:** Mustafa Arda Onar, Mehmet Selim Nural, Aydın Deveci, Bilge Can Meydan

**Affiliations:** 1 Bartın State Hospital, Bartın, Turkey; 2 Ondokuz Mayıs University, Samsun, Turkey

**Correction to: Abdominal Radiology (2026) 51:3–13**

**https://doi.org/10.1007/s00261-025-05043-2**

The original version of this article contained error in Abstract section.

In Abstract under “Materials and Methods” heading, the references were included erroneously and it should be removed.

For completeness and transparency, the both incorrect and correct Abstract sections are displayed below.

**Incorrect Abstract:**

**Introduction** This study evaluates the effectiveness of virtual magnetic resonance elastography (VMRE), a new diffusionweighted imaging (DWI)-based method, for detecting liver fibrosis, comparing it with the more accessible ultrasound elastography (USE).

**Materials and methods** This prospective study enrolled patients with chronic liver disease who were referred for liver biopsy. Inclusion criteria were: Sepanlou (Lancet Gastroenterol Hepatol 5:245–266, 2020) clinical indication for liver biopsy and Rinella (Journal of Hepatology 79:1542–1556, 2023) eligibility for MRI. Exclusion criteria included: Sepanlou (Lancet Gastroenterol Hepatol 5:245–266, 2020) MRI contraindications, Rinella (Journal of Hepatology 79:1542–1556, 2023) hepatic iron overload, D’Amico (Hepatology International 12:34–43, 2018) clinical or laboratory evidence of acute hepatitis or cholestasis, and Terrault (Hepatology (Baltimore, Md) 63:261, 2016) inadequate image quality (motion artifacts, low signal-to-noise ratio). All patients underwent 3 T VMRE (b = 200/1500 s/mm^2^) and two-dimensional shear wave elastography (2D-SWE). VMRE was analyzed by two blinded readers; USE by a single radiologist. Using METAVIR staging (F0-F4) as reference, fibrosis was categorized as F0-1 vs. F2-4 and F0-2 vs. F3-4. Statistical analyses included ICC, Bland–Altman, Kruskal–Wallis with Bonferroni-corrected Dunn tests, and ROC analysis. An HBV subgroup (n = 33) and a non-HBV group (n = 16; including metabolic dysfunction-associated steatotic liver disease (MASLD), autoimmune, and toxic hepatitis) were analyzed separately to assess VMRE performance across different etiologies.

**Results** Initially, 59 patients were enrolled. After excluding 10 patients due to MRI contraindications, hepatic iron overload, or inadequate image quality, 49 patients were included in the final analysis (mean age 48.2 ± 14.9 years; 28 males, 21 females; 67% HBV-positive). VMRE demonstrated significant limitations in clinical utility, failing to discriminate fibrosis stages in the overall cohort (AUC 0.45–0.51, p > 0.05). While HBV-infected patients showed some promise with an overall significant variation across stages (p = 0.004), post-hoc analysis revealed VMRE could only distinguish between the extreme ends of the fibrosis spectrum (F0 vs. F4: adjusted p = 0.0058). This restricted diagnostic capability was reflected in the HBV subgroup’s modest AUC values of 0.75–0.76, which remained below clinical acceptability thresholds. In striking contrast, ultrasound elastography exhibited robust performance across all analyses. It achieved excellent diagnostic accuracy (AUC 0.86–0.95) with highly significant p-values (< 0.001) for all fibrosis classifications, along with clinically practical threshold values (8.85–10.1 kPa). Inter-rater agreement for VMRE was excellent (ICC = 0.972), and intra-rater agreement for USE was good (ICC = 0.756).

**Conclusion** VMRE demonstrates insufficient diagnostic accuracy for fibrosis staging in both HBV and non-HBV populations, with only limited ability to distinguish extreme fibrosis stages (F0 vs. F4) in HBV patients. While showing excellent technical reproducibility (ICC = 0.972), its poor discriminative performance (AUC 0.45–0.76 across groups) and inability to differentiate intermediate stages preclude clinical utility. In contrast, USE achieved consistently superior diagnostic accuracy (AUC 0.86–0.95) with practical threshold values, supporting its preference over VMRE especially in centers lacking access to standard MRE. Further VMRE development requires technical optimization and larger validation studies.

**Correct Abstract:**

**Introduction**: This study evaluates the effectiveness of virtual magnetic resonance elastography (VMRE), a new diffusion-weighted imaging (DWI)-based method, for detecting liver fibrosis, comparing it with the more accessible ultrasound elastography (USE).

**Materials and Methods**: This prospective study enrolled patients with chronic liver disease who were referred for liver biopsy. Inclusion criteria were clinical indication for liver biopsy and eligibility for MRI. Exclusion criteria included MRI contraindications, hepatic iron overload, clinical or laboratory evidence of acute hepatitis or cholestasis, and inadequate image quality (motion artifacts, low signal-to-noise ratio). All patients underwent 3 T VMRE (b = 200/1500 s/mm^2^) and two-dimensional shear wave elastography (2D-SWE). VMRE was analyzed by two blinded readers; USE by a single radiologist. Using METAVIR staging (F0-F4) as reference, fibrosis was categorized as F0-1 vs F2-4 and F0-2 vs F3-4. Statistical analyses included ICC, Bland–Altman, Kruskal–Wallis with Bonferroni-corrected Dunn tests, and ROC analysis. An HBV subgroup (n = 33) and a non-HBV group (n = 16; including metabolic dysfunction-associated steatotic liver disease (MASLD), autoimmune, and toxic hepatitis) were analyzed separately to assess VMRE performance across different etiologies.

**Results**: Initially, 59 patients were enrolled. After excluding 10 patients due to MRI contraindications, hepatic iron overload, or inadequate image quality, 49 patients were included in the final analysis (mean age 48.2 ± 14.9 years; 28 males, 21 females; 67% HBV-positive). VMRE demonstrated significant limitations in clinical utility, failing to discriminate fibrosis stages in the overall cohort (AUC 0.45–0.51, p > 0.05). While HBV-infected patients showed some promise with an overall significant variation across stages (p = 0.004), post hoc analysis revealed VMRE could only distinguish between the extreme ends of the fibrosis spectrum (F0 vs F4: adjusted p = 0.0058). This restricted diagnostic capability was reflected in the HBV subgroup's modest AUC values of 0.75–0.76, which remained below clinical acceptability thresholds. In striking contrast, ultrasound elastography exhibited robust performance across all analyses. It achieved excellent diagnostic accuracy (AUC 0.86–0.95) with highly significant p-values (< 0.001) for all fibrosis classifications, along with clinically practical threshold values (8.85–10.1 kPa). Inter-rater agreement for VMRE was excellent (ICC = 0.972), and intra-rater agreement for USE was good (ICC = 0.756).

**Conclusion**: VMRE demonstrates insufficient diagnostic accuracy for fibrosis staging in both HBV and non-HBV populations, with only limited ability to distinguish extreme fibrosis stages (F0 vs F4) in HBV patients. While showing excellent technical reproducibility (ICC = 0.972), its poor discriminative performance (AUC 0.45–0.76 across groups) and inability to differentiate intermediate stages preclude clinical utility. In contrast, USE achieved consistently superior diagnostic accuracy (AUC 0.86–0.95) with practical threshold values, supporting its preference over VMRE especially in centers lacking access to standard MRE. Further VMRE development requires technical optimization and larger validation studies.

The original article has been corrected.

